# Human Attention Restoration, Flow, and Creativity: A Conceptual Integration

**DOI:** 10.3390/jimaging10040083

**Published:** 2024-03-29

**Authors:** Teresa P. Pham, Thomas Sanocki

**Affiliations:** 1 Department of Psychology, University of South Florida, St. Petersburg, FL 33701, USA; tpp1@usf.edu; 2 Department of Psychology, University of South Florida, Tampa, FL 33620, USA

**Keywords:** attention, executive attention, attention restoration, attentional ecology, nature, flow, creativity

## Abstract

In today’s fast paced, attention-demanding society, executive functions and attentional resources are often taxed. Individuals need ways to sustain and restore these resources. We first review the concepts of attention and restoration, as instantiated in Attention Restoration Theory (ART). ART emphasizes the role of nature in restoring attention. We then discuss the essentials of experiments on the causal influences of nature. Next, we expand the concept of ART to include modern, designed environments. We outline a wider perspective termed attentional ecology, in which attention behavior is viewed within a larger system involving the human and their interactions with environmental demands over time. When the ecology is optimal, mental functioning can be a positive “flow” that is productive, sustainable for the individual, and sometimes creative.

## 1. Introduction

The relation between humans and the environment has undergone drastic change, from urbanization and environmental considerations to technological advancements and the explosion of digital worlds. Technical advances bring with them an increasingly complex myriad of stimulation that demands attention, such as busy city traffic, flashy advertisements, and a variety of notifications designed to distract. Attention is necessary for a human to survive in this sea of stimulation; attention is needed to set goals, select information, and inhibit distractions [1,2]. The constant use of attention leads to attentional fatigue [3,4]. This, in turn, causes negative outcomes such as difficulties with focusing and planning, and susceptibility to distraction [4,5]. Thus, it becomes important to consider the idea of attention restoration—ways in which we can restore and better sustain attentional functioning [4]. 

In this paper, we discuss the theory and concepts of attention and restoration [4], and then expand the theory within a larger perspective that includes behavior over time, as well as modern designed environments. We synthesize past research perspectives that explored topics of executive demands, the restorative properties of nature, and the flow state into a larger perspective we call attentional ecology. Ecology emphasizes the health of a larger system involving the human and their interactions with the plethora of environmental stimulation and demands over time. When the ecology is optimized, mental functioning can be a positive “flow” that is productive and sustainable for the individual, and even creative [6]. Attentional ecology can help us combine nature, modern technology, and our individual abilities to better survive in the changing world. (The term “ecology of attention” was introduced by Citton in 2017 [7], within a wider perspective that included group behavior and cultural theory. These broader perspectives are important and complement the present emphasis. We focus on the topic of mental behaviors and their relation to the environment).

Before we begin, we note that attention refers to a complex set of mental functions that are still being defined (see [1,2]). Researchers generally agree that important functions of attention include selecting and prioritizing information and inhibiting distractions. Attention can also be viewed as a resource that is scarce; when attention is depleted (fatigued), attentional functioning is degraded. We begin with “high-level” attention, which includes executive functions such as interconnecting modalities and brain regions, setting main goals, and assembling new ideas [2,8,9,10,11]. 

## 2. Executive Demands, Problem-Solving, and Fatigue 

In general, executive attention prioritizes perception and cognition [1]. Executive functions help us attend to our environment, interpret and work with complex ideas, and regulate our behaviors [12,13]. When demands in the environment are high, such as having to cross a busy street while inhibiting nearby distractions, the control functions of executive attention become critical. Not surprisingly, in today’s fast-moving world, executive attention is often overused. Nevertheless, it is related to life successes including academic achievement, job performance, well-being, and healthy aging [13,14].

Executive attention is important for problem-solving and creativity. When working on a new problem, the individual usually activates potentially relevant information in their working memory while avoiding distracting or irrelevant information. More generally, attention must be flexible, allowing the individual to skillfully juggle new and old information and change focus [15]. Attention is more likely to be facile when rested. A critical stage of innovation and problem solving is the creative insight, in which new combinations of ideas are brought together in the inventor’s working memory [16,17]. Insight is more likely when attentional skill is maximized. When the mind is stressed or taxed, creative performance drops [16].

Cognitive fatigue and decreased executive attention can lead to undesired outcomes. Fatigued participants show difficulties in focusing and planning [5]. Consequently, depletion of attention resources can increase the risk of errors occurring during tasks, such as forgetting important meetings or sending the wrong documents. In experimental research, participants who completed a visual attention task for three hours straight had increased difficulties staying alert and maintaining their attention, leading to more errors, false alarms, and missed targets [18]. Mental fatigue can also increase drowsiness and decrease alertness in drivers, resulting in traffic accidents when driving [19]. Cognitive fatigue even affects moral reasoning [20] and is related to judges making stricter parole decisions at the end of a session compared to the beginning of a session or after a break [21]. Thus, the issue of depleted attentional resources affects all individuals at various levels of their everyday lives. 

Because of the general importance of executive processing, government and public health efforts have targeted executive processing for strengthening, along with cognition and brain health in general [14,22]. Given that executive processing is applied to demanding situations and subject to fatigue, the idea of training the brain through mental exercise provides a possible mechanism to increase brain health [23,24]. Brain training could complement the usual health behaviors such as physical exercise, good sleep and diet, stress management, and social engagement [25]. However, while brain-training efforts are often effective for increasing performance on the trained task (“near transfer”), it is not clear whether brain training impacts brain health in a general way (“far transfer”). The holy grail of far-transfer remains elusive—brain training may not benefit the wide range of tasks beyond the trained tasks, including executive attention in general [23,24]. 

In fact, one can ask if there are any interventions that improve mental functioning in a general way? Time in good nature has long been viewed as such an intervention. The early psychology theorist Jane Austen wrote, “To sit in the shade on a fine day and look upon verdure is the most perfect refreshment”. Hippocrates advocated that, “Nature itself is the best physician”. Throughout time, individuals, groups, and even movements like Romanticism have cited the healing power of nature, in which a return to nature serves as an antidote to issues that arise in the city. Nature being restorative to the mind, spirit, and body has been an idea relevant across multiple disciplines, including urban planning [4], biology [26], literature and the arts (Romanticism), and meditation [27]. 

The idea of the restorative powers of nature has inspired research on its benefits. In the 1970s, Kaplan and Kaplan [4,28] began studying human preferences among scenes depicted in photographs, including scenes with more or less nature. Finding that scenes with nature were generally preferred, Kaplan and Kaplan [4] extended their research to test for restorative effects, initiating a body of research on nature and its role in attention restoration. 

## 3. Nature and Restoration 

Kaplan and Kaplan [4] conceptualized their research as a study of the relation between nature and human activity; it is this relation, not nature itself, that is restorative. Integrating over two decades of research insights, Kaplan and Kaplan [4] concluded that nature has many positive influences, and that the most important influences can be understood in terms of mental processes that can occur after fatigue has set in. Mental fatigue results from high-effort work—both reasonable effortful work over longer time periods, and unpleasant work which may seem effortful even after brief periods. Fatigue can also result from repeated actions in confusing or stressful environments, such as crossing hectic city streets. Kaplan and Kaplan [4] argue that the experience of nature can provide restoration, often at relatively little cost. A walk through a nature park or garden is perhaps the classic brief restorative experience. However, longer experiences such as a vacation in nature can be even better. The strongest experiences, such as camping alone in the backcountry, may be the most restorative. Kaplan and Kaplan studied the influence of extreme Outward-Bound experiences, a program in which adolescents take a trip in nature and learn to survive on their own. They found that the program can result in lasting positive changes in the self, such as self-confidence [4]. Based on this body of research, Kaplan and Kaplan proposed Attention Restoration Theory (ART). 

**Figure 1:**
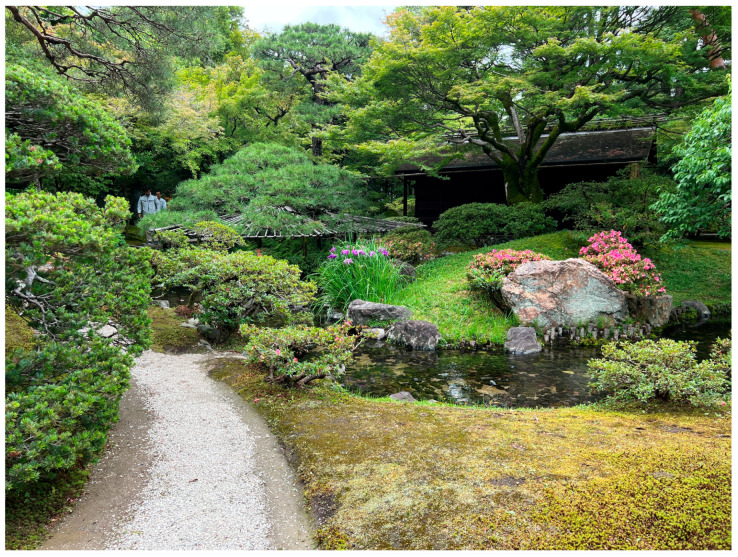
Garden path.

ART—see also Kaplan [29,30]—explains the aspects of nature that give it the power to refresh minds and restore fatigue. According to ART, nature has four inherent properties that contribute to restoration, regardless of whether actual travel through nature occurs: being away, extent, compatibility, and soft fascination. The properties begin with being away, which refers to being psychologically detached from present demands. Extent refers to immersion in a larger environment that a person can relate to; this can be a real, present environment, or metaphysical connections to a world beyond. Compatibility refers to an environment that is consistent with an individual’s preferences for activity; the ability to do what one wants to. The last property is somewhat complex; it is soft fascination, caused by stimuli and environments that are inherently interesting, but not demanding. Soft fascination is a mode of attention that is quite different from rapidly responding to urgent demands. For example, a nature garden is full of lovely flowers, plants, and arrangements, and little mental effort is needed to appreciate it beyond seemingly effortless natural actions such as walking and looking (Figure 1). Following ART, all these properties contribute to the overall restorative effects of nature on the mind. 

According to ART, exposure to nature allows attentional resources to be restored because it changes the predominant mode of attention. In everyday life, one often uses an effortful subset of executive attention: directed, or voluntary, attention. Directed attention produces focus while suppressing distracting stimuli. The inhibition of other stimuli (which may be more interesting than a mundane relevant task) is a primary function of directed attention. When directed attention is working well, we do not notice distractions. However, the constant use of directed attention depletes mental resources and leads to fatigue and decreased cognitive performance [3,31]. This fatigued state may also involve pain (feeling tired and “sore”). And it contributes to “crankiness” in a busy city because there are less attentional resources to inhibit anti-social thoughts and actions [4].

The restorative attentional mode is soft fascination, which is a form of involuntary attention or effortless attention. In this mode, directed control is not necessary because factors such as a “nice” environment can guide behavior—little effort is needed to see the flowers. The pathways of a nature park guide navigation pleasantly, and the individual is free to get lost in their thoughts. There are no urgent demands. When involuntary attention dominates, directed attention can replenish [4,31,32]. Pleasant internal behaviors such as reflection and fantasy can occur, while directed attention rests [4]. In essence, ART posits that by being in a safe natural environment, individuals can freely engage in effortless attention, allowing directed attention to replenish and restore. As a result, their mental powers are renewed and can flourish. 

### 3.1. Testing Attention Restoration Theory with Experiments 

Does time in nature actually cause restoration? Experiments support this idea, but there are interesting complexities. Experiments generally follow a three-phase procedure. The first phase involves a repeated task that fatigues attention and a measure of attentional power; these two functions are typically combined in a difficult attention task. The second phase is a nature experience or a control experience. Lastly, attentional power is measured again. Thus, in a classic experiment, Berman et al. [32] first measured and fatigued attention with a backward digit span task (detailed in Section 3.2 Replicability, below). Then, some subjects walked through a nature park (the university arboretum) while other subjects walked down a busy city street. Attention was measured again, and there was a reliable increase in power for the nature walkers. In general, this result has been replicated in numerous studies, differing in many details [33,34,35]; for meta-analyses, see [36,37]. There can also be positive effects on mood and stress levels when measured, but these effects appear to be independent of the mental benefits rather than causative [38]. 

We agree with the conclusions of these researchers, that nature experiences can cause restoration. Interestingly, the effective nature experiences can be virtual rather than actual, (e.g., Berman et al.’s second experiment) [32]. However, the effects of nature experiences are not always strong and can be difficult to replicate. The actual restoration effect is likely to be small in comparison with other sources of variation: differences between individuals in preference, differences that arise as a new task is learned, and differences in individual experience. These sources of variation produce “noise” that competes with the test signal of restoration. Further research is needed, in our view, to take ART research beyond its adolescent stage. We now delve into two essential issues—replicability, and meaningful effect size. 

### 3.2. Replicability 

Replicability refers to whether similar positive restorative effects are likely to be found in a new, different experiment. Because publication bias favors positive results, experiments that work (restore) are more likely to be written up and published than experiments that do not. A direct method for examining replicability is to repeat the experiment in related forms, and to report all of the experiments’ results. Stenfors, Berman and colleagues [38] were able to do so with 13 experiments that all used the same measure of attentional power—the backward digit span (BDS). The combined results show that restoration effects occur, and they provide a high-powered estimate of the size of the restoration effect. However, the effects of learning and practicing the attention task (BDS) are larger than the restoration effect, especially in the first parts of an experiment. Let us delve into the relevant complexities. 

In restoration experiments, a sensitive design must be used. The preferred design involves repeated testing of the same participants—having the same people experience both nature and city conditions at different times (typically separated by 2 weeks). Complications arise because attention tasks are difficult, and participants improve on the task over time. For example, the most-used task is the BDS task, a classic working memory task that challenges individuals to encode a string of digits, maintain them in memory, and then actively process them by repeating them back in reversed order. The number of digits begins at two and gets longer after each correct trial. Attention power (the “span”) is defined by how many digits an individual can handle, which is typically about eight. The task has multiple component processes in which an individual can improve over time and become better coordinated. In the typical sensitive design, each participant will engage in four sets of BDS trials and improve substantially across the total experiment. Therefore, the experimental results have overlapping influences, which can obscure restoration effects. The best estimate of the restoration effect comes from the second session, which is still balanced between nature and the city, but with the practice effects leveled out. 

### 3.3. How Large Is the Restoration Effect?

Based on their 13 experiments, Stenfors et al. [38] gauged the restoration effect to be an increase in BDS digits of 0.51 after nature experience, compared with a decrease after city experience (of −0.23 digits). This is a total gain, caused by nature experience, of 0.74 digits. Participants were able to encode and process more information during a single trial. But what does this mean, beyond an increase of three quarters of a digit? 

### 3.4. Beyond Digits: Creative Cognition 

If the mind is truly “restored”, the consequences of restoration should extend beyond working memory of digits to more complex and interesting aspects of cognition. Restoration should help thinking become more productive, and perhaps more fluid and creative over time. In fact, there is initial evidence indicating that extreme nature experience results in a marked increase in creativity [39]. Furthermore, because thinking continues over time, restoration could compound itself—an initial boost could start more positive thinking, which in turn leads to more and better thinking, and good ideas could feed more thinking. Thus, with continued good conditions, 0.74 digits could sometimes lead to profound benefits such as new ideas and breakthroughs. Leading theorists have suggested the processes by which this can occur [31,40,41]. To illustrate these processes over time, we invite the reader on a walk in nature:

After hours of hard work, one closes the outer office door. New stimulation will be available, but work-related thoughts may not stop immediately because of task inertia [42]. Nevertheless, work thoughts can eventually slow down, and better thinking can begin upon entrance to a nature park. The body begins to relax with continuous easy walking (a physiological effect and a creative boost) [43]. Flowers and fluffy clouds can be noticed but they pose no demands; attention is free to wander. One can enjoy scenery, but attention can also turn inward. The most general effect of restoration is a general quieting of the mind, because the “noise” of competing thoughts begins to subside. Gradually, attention can replenish and play a more skillful role in thinking, at a slower and more relaxed pace. Mind wandering is generally an effortless inward activity that allows for more restoration and possibly creativity. The individual can better focus on priorities and better enjoy the day.

Some individuals may even return to work mentally, but in a more productive way. Reflective processing can occur: reviewing the day, identifying hidden issues, and separating more essential thoughts from the noise. There may have been an interesting but unsolved problem. The individual can return to it, which involves re-activating information in working memory. This is a dynamic and skillful process, also called “memory retrieval” in a large literature [44]. However, because the restored mind can work more skillfully and at a slower pace, memory activation can be better coordinated. Furthermore, even brief periods away from a problem would allow unconscious processes to restructure memory, also contributing toward a solution [45]. These compounding benefits can increase the probability of creative insight.

This example illustrates three general advantages of the restored mind. First, internal noise is quieted, so there is less competition for thought [4]. Second, attention can become stronger, increasing the skill of attentional control. Third, a host of other beneficial processes—incubation, reflective thought, and mind wandering—can flourish in a safe environment [31,40,41]. These processes are compatible with each other and can compound the positive effects of restoration as the walk continues. The compounding positive of effects over time begs for a wider perspective. 

## 4. Attentional Ecology 

**Figure 2:**
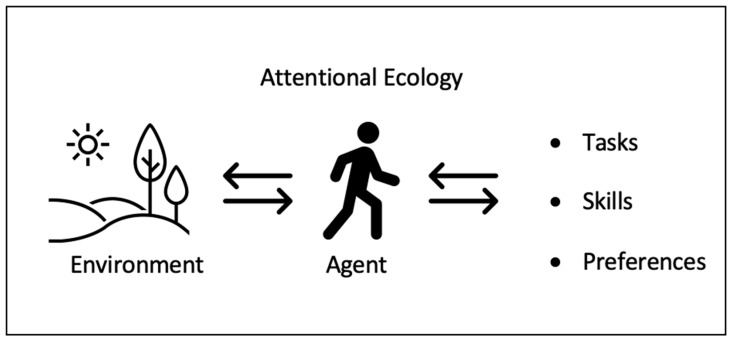
Visual representation of attentional ecology perspective.

Attentional ecology is a larger, more systemic perspective on attention and mental processes over time. The attentive person is an agent functioning within an environment (Figure 2). When the relation between attention and environment is healthy (e.g., attention can stay strong rather than being taxed), attentional skill can be sustained over time. The state of attention depends on the larger context of activity, which includes scheduling. If there is a large and complex task to be completed (requiring hours of directed attention), breaks can help sustain quality effort. Concepts such as soft fascination can be applied in small ways to sustain attention. Bad environments (loud noise, interruptions, threats) will tax attention rather than sustain it, and reduce the efficiency of thought. Such ideas are understood implicitly by effective workers. The concept of attentional ecology may help to make the discussion more explicit (see also [7]). 

ART and attentional ecology are related to other large issues and literature. Numerous studies have explored the scheduling and sharing of attention, including primary tasks, secondary tasks, and task switching and task threading [2,46,47]. One issue related to time is rest, and there is a large body of research that focuses on work and rest [48]. It shows that the human mind must take breaks and rest; an individual cannot continually attend at the highest level of efficiency [49]. 

Other important issues in attentional ecology involve physical and mental safety. Participants in experiments can assume that they are safe: their walk in a nature park will have a clear and safe trail, and be free of hungry wolves, extreme weather, or unreasonable demands. However, in both cities and rural communities, many individuals do not have access to physically safe walking routes, making restoration much more difficult. Additionally, mental safety can be threatened for people working online, who face increasing security and privacy concerns. 

Attentional ecology is a broad perspective in the emerging culture of attention. It is related to the concept of the attention economy [50]. However, ecology emphasizes health, and in this case the mental health of the individual and the environment they exist in. The human is an agent in their ecology, someone who can influence their own mental health and be influenced by their environment in return. Individuals can use personal preferences to guide themselves toward a restorative environment. An optimal ecology is similar to the flow state [6]. In flow, individuals can maintain their attention at a healthy level because their individual skills align with the task and environment at hand. The concept of attention economy implies that the agent can sometimes decide how to spend their attention, but this sense lacks reference to the health of the situation, which we emphasize. Attention economy can also refer to the commercial competition for human attention, which can be exploitive. Social media use is associated with significant mental health problems in younger people, probably because of its exploitive design [51]. Healthy mental development can be encouraged by an ecology designed from a humane perspective [52,53].

Attentional ecology can help people deal with new technologies. Before smart technology and its numerous alerts and notifications, a worker focusing on a large task could become immersed in the task and stay focused on it. Rarely would the worker intentionally switch attention away from the task, much less constantly switch their attention back and forth. There are costs for switching to a secondary task, and for re-starting the primary task [46]. Thus, notifications and alerts serve as secondary tasks that tax attention. Sometimes, the secondary task can impose an affective threat to a person, including their sense of self. 

## 5. Science Experiments Are Impoverished, but Personal Experiments Are Not

Because of the critical need for experimental control, restoration experiments are impoverished. The experiments are usually “one shot,” rather than examining cognition over time. The experiments also ignore basic factors like fun or affect. The controlled nature of ART experiments thus could miss out on the potential role of individual preferences and context in restoration. Personal experiments, on the other hand, can be less impoverished. An individual can optimize restoration by compounding positive factors. The nature walk may be more restorative with an ice cream cone or one’s favorite beverage. Happy people in a park can add positive social factors. Each person can design their own experimental conditions. The idea of attentional ecology addresses the idea that an individual’s attention can be influenced by both their individual preferences and their surrounding environment. Individuals can work towards maintaining their attention through flow, which depends on an alignment of individual skills, interests, and task challenges. This next section connects flow to ART and attention ecology.

## 6. The Flow State

Flow may play a key role in healthy attention. Flow is the feeling of giving one’s full attention to an activity in such a focused and immersive way that outside distractions disappear—the individual becomes fully aware of the present moment [6]. Flow is based in an individual’s specific interests and skills, in contrast to restoration, which has usually been thought of and researched as a general process across individuals.

The flow state is often described in sports or creative pursuits. For example, a songwriter may become so immersed in their creative process that five or six hours pass without notice, or a basketball player making a practiced shot will experience the coordinated flow of that action.

Flow is similar to soft fascination in that both require the individual to engage in an activity within an optimal range of challenge; the challenge should be intrinsically meaningful and permit the feeling of control. However, unlike ART’s emphasis on nature experience, flow emphasizes strong concentration and focused attention towards a specific task of one’s choosing. There is little anxiety during flow; the individual’s skills and knowledge are aligned with the task at hand [54]. Unlike restorative walks in nature, which tend to focus on activities disengaging enough to provoke introspection, the flow state occurs during focused activities that are familiar and stimulating.

Individuals can benefit by finding flow during cognitively demanding activities. This could occur through mindfulness—the awareness that arises from being in the present moment without resistance or attention to other stimuli like stressful thoughts or future/past oriented thinking [55]. If the flow state is achieved, individuals may be less prone to burnout and attention fatigue [56]. Flow could be a more sustainable form of directed attention, causing inherent pleasure, and resulting in longer work periods before fatigue occurs.

Between restoration and flow may lie a middle ground called effortless training, or effortless attention. Effortless attention is the idea that actions or skills that involve modest mental effort can enhance cognition and performance in ways different from effortful training with cognitively demanding tasks [24]. Effortless attention involves parasympathetic nervous activity—the part of the nervous system that handles rest, digestion, and modest sensory pleasure. This contrasts with the sympathetic system, which excites fight and flight [24].

Effortless attention also activates the default mode network (DMN) of the brain, which is associated with introspective thought and reflection [31,57]. Tang and co. [24] designed an effortless regimen of integrative body–mind training; the method focuses on being in a state of restful alertness, allowing thoughts to flow naturally while being aware of one’s body, breathing, and external environment. One to four weeks of training strengthened the posterior cingulate cortex, a hub of the DMN that is associated with attention allocation, adaptive behavior, and cognitive processes [24].

Thus, effortless training of attention through methods like nature experience and flow can serve as brief interventions for improving executive attention and cognitive functioning. By melding ideas of nature restoration, mindfulness, and flow, individuals may be able to increase their productivity, concentration, and creativity, while decreasing mental fatigue and improving brain health.

## 7. Where ART Meets Design

Can we apply what we know of nature’s restorative properties to encourage attention restoration through other mediums? Because of climate challenges and the development of more than 50% of the world’s population now living in urban areas [58], it is becoming more difficult to find easily accessible, good nature areas. This makes it critical for individuals to find other ways to restore their cognitive resources.

Urban designers support attention by incorporating green spaces and nature in cities, including parks and natural landscaping, and windows with good views [4,59,60]. Incorporation of restorative nature elements through technology and virtual reality has also been considered, with researchers finding that virtual natural environments can be restorative and enhance mood while also decreasing stress [61,62]. Urban design and technologies could integrate the knowledge of restorative nature properties and the idea of attentional ecology. We now explore whether cultural developments such as art, video games, and museums can serve as restorative experiences.

### 7.1. Visual Design: Art, and Video Games

Past studies found that viewing pictures of nature improves attention [32,34], which begs the question of whether similar kinds of visual stimuli can produce restorative experiences for individuals. ART proposes that four critical elements to attention restoration are soft fascination, being away, extent, and compatibility. Art and/or video games could fulfill the four elements critical to attention restoration: soft fascination (if they involve modest-attention-demanding tasks that are interesting to the individual), being away (individuals can psychologically detach from present worries through immersion in the work), extent (immersion in a larger virtual world), and compatibility (individuals can choose their own preferred artwork or game). In fact, the restorative experiences of nature and related concepts found in nature and art/video games (e.g., visuals, sound) may be connected through the idea of awe [63]. Awe is a feeling that emerges from encountering something that is so vast, it is difficult to perceive without accommodating current knowledge structures; for example, a person may experience awe when witnessing a solar eclipse. Awe can arise through experiences with nature, visual art, and music. Positive experiences of awe are linked with positive mental and physical outcomes such as improved well-being, decreased stress and anxiety, and longevity [63]. Thus, awe could be a contributing factor underlying potential restorative benefits of nature and mediums beyond nature, like art and video games. Video games, specifically, have the potential to combine all these aspects: incorporating virtual nature, music, awe, and design to create an immersive, restorative experience for individuals.

Some video games are designed with the goal of improving mental health; for example, Gris, which explores and leads the player through the stages of grief with its beautiful watercolor artwork and design [64], and Stardew Valley, a game designed to help players with depression [65,66]. Thus, it may be possible to use visual media like video games or art to aid with attention restoration and mental fatigue. These mediums can incorporate research and interdisciplinary design to create research-based mediums to promote mental health.

### 7.2. Museums

Museums could be a great avenue for restorative environments; they are places that are explicitly designed with the intent of preserving and sharing knowledge while also providing the guests with a reflective and humbling experience. In fact, the museum effect is the idea that museum visits can evoke complex, powerful, and unforgettable experiences for their visitors [67]. Museums can serve as spaces that provoke feeling of nostalgia, learning, and past–present resonance. Using artifacts of the past and present while encouraging reflective thought can promote introspection from the guests and a sense of awe. Wong et al. [68] found that virtual tourist experiences were restorative because they evoked feelings of immersion, touristic learning, and nostalgia. Museums also allow individuals to stroll through exhibits at their own pace and choose which artwork to contemplate, depending on preferences. Designed as safe spaces, museums can act as a non-distracting environment that allows viewers to immerse themselves in the history, art, or science that it presents.

Designers of museums increasingly incorporate elements of nature into the buildings. The Metropolitan Museum of Art (MET) in New York City, for example, includes exhibition areas that double as rest areas—the Temple of Dendur stands in an open room with sunlight from the northeast windows and is placed near an artificial river area that mimics the Nile. Similarly, the MET’s Astor Chinese Garden Court serves as a model of a scholar’s court from the Ming dynasty; like the original court’s design, it incorporates natural elements such as plant, sunlight, and running water, arranged in accordance with Feng Shui. The area of the museum serves multiple purposes as an exhibition, work of art, rest area, and place of learning.

Beyond nature, designers of museums can opt for intentional, restorative experiences through incorporation of technology and perception, inspired by the recent trend of interactive perception/art experiences that fuse technology, design, and perception/cognition. For example, AREA15 in Las Vegas is an experiential art complex that allows visitors to experience art galleries through immersive technology that includes 3D projection mapping, holography, and more [69]. Superblue in Miami, Florida, promotes art experiences in which visitors walk through and interact with art structures through sight, hearing, or even taste [69].

## 8. Final Thoughts

In today’s fast paced, attention-demanding society, executive functions and attentional resources are often taxed. Individuals need to find ways to sustain and restore these resources. ART advocates using properties of nature to restore directed attention and cognitive performance in general, including creativity. Beyond ART, individuals may be able to better sustain attention by tapping into the flow state of effortless attention. These effects may extend beyond general performance and suggest a larger system of attention and mental processes over time. Conceptualizing attention as an ecology allows us an insight into how individuals can restore and sustain attention through interaction with their environments and understanding themselves. Future research could explore the connections between the individual as an active agent in their environment and the restorative properties of the environment around them.

In the meantime, it is still important to support brain-healthy attentional ecology. Executive functions can be kept effective through a combination of good lifestyle choices and a balanced attentional ecology. Each individual should arrange their ecology in a way that best works for them. Video games and art may be mediums in which individuals tap into effortless attention and enhance their cognitive performance. Beyond the individual level, large scale designs with nature in mind, such as museums, could serve to further enhance attention. Designs could act as avenues for mental travel over time; travel that helps attention replenish and flourish.
